# Case Report: a novel variant in *WT1* leads to focal segmental glomerulosclerosis and uterovaginal anomalies through exon skipping

**DOI:** 10.3389/fneph.2025.1542475

**Published:** 2025-04-01

**Authors:** Jonathan Marquez, Lauren O’Sullivan, Audrey E. Squire, Ginny L. Ryan, Katherine E. Debiec, Anne-Marie Amies Oelschlager, Margaret P. Adam

**Affiliations:** ^1^ Department of Pediatrics, Division of Genetic Medicine, University of Washington and Seattle Children’s Hospital, Seattle, WA, United States; ^2^ Department of Obstetrics and Gynecology, Division of Reproductive Endocrinology and Infertility, University of Washington, Seattle, WA, United States; ^3^ Department of Obstetrics and Gynecology, Division of Pediatric and Adolescent Gynecology, University of Washington and Seattle Children’s Hospital, Seattle, WA, United States

**Keywords:** podocytopathy, nephrotic syndrome, kidney transplant, renal genetics, splicing, WT1

## Abstract

**Background:**

Podocytopathies are a varied set of renal diseases in which podocytes are unable to perform their typical filtration function within the glomerulus. This typically leads to edema, proteinuria, and hypoalbuminemia early in life. Among podocytopathies, focal segmental glomerulosclerosis (FSGS) is characterized by histology demonstrating segmental and focal sclerosis of the glomerular tuft. FSGS affects an estimated 1–20 per one million individuals and leads to significant morbidity and mortality related to renal failure. While FSGS can be attributed to many causes, such as drug reactions and infections, underlying pathogenic genetic variants play an increasingly well-recognized role in this disease.

**Case:**

A 38-year-old 46,XX female patient of self-reported Cambodian ancestry was evaluated due to her history of atypical uterovaginal morphology. She had a history of hypertension and nephrotic range proteinuria that was diagnosed early in adulthood. A kidney biopsy at that time revealed FSGS. Following worsening renal function and subsequent end-stage renal disease (ESRD), she underwent a kidney transplant at 33 years of age. After kidney transplant, she presented with hematocolpos and was found to have distal vaginal atresia and an arcuate uterus. She underwent vaginoplasty and then had regular menses. She was noted to have persistently elevated follicle stimulating hormone levels, consistent with primary ovarian insufficiency, but with normal anti-Müllerian hormone levels. Assessment of her family history was suggestive of other individuals in her family with similar renal disease and uterine differences. Genetic analysis identified a *WT1* variant (c.1338A>C; p. =) of uncertain significance that is also present in her similarly affected mother. To help clarify the potential impact of this variant, we completed a mini-gene assay to detect *in vitro* splicing changes in the presence of the *WT1* variant sequence uncovered in this individual. This demonstrated resultant aberrant splicing that further supports the pathogenicity of the uncovered variant for this individual.

**Conclusions:**

To our knowledge, this represents the first case of a podocytopathy with co-occurring uterovaginal anomalies due to exon skipping in *WT1*. The patient exhibited a severe course of chronic kidney dysfunction requiring a kidney transplant. Clinical RNA sequencing to clarify variants impacting splicing remains challenging due to tissue- specific gene expression for genes such as *WT1*, thus, research-based assays may be beneficial to understand the consequence of rare or previously uncharacterized variants.

## Introduction

Podocytopathies are a group of diseases in which renal glomerular filtration functions are compromised. This causes proteinuria, edema, and typically leads to chronic kidney dysfunction over time as part of nephrotic syndrome (1–3). While podocytopathies leading to nephrotic syndrome may respond to steroid-based therapies, those that are unresponsive to these limited medical interventions are termed steroid-resistant nephrotic syndrome (SRNS) and invariably require renal dialysis and/or renal transplantation for an individual to survive (4). Podocytopathies are the most common cause of end-stage renal dysfunction early in life (5). When nephrotic syndrome is unresponsive to steroid therapy, renal histology reveals focal segmental glomerulosclerosis (FSGS) or diffuse mesangial sclerosis (6, 7). These are signs of irreversible glomerular damage. Pathogenic variants in nearly 100 genes have been described to lead to monogenic SRNS (8–10). Podocytes appear to be the critically impacted sites of dysfunction in SRNS (2). Many studies that have uncovered pathogenic variants leading to SRNS have illuminated the role of the proteins encoded by these genes in signaling pathways and structural components important for podocyte establishment, function, and morphology (11–15). As genetic testing is more frequently deployed as part of the assessment of podocytopathies, monogenic causes of the disease can be identified in approximately 30% of affected individuals (16, 17).

Wilms’ tumor suppressor gene 1 (*WT1*) is one of the most highly expressed genes in both mouse and human podocytes and demonstrates limited expression in other tissues (18, 19). Pathogenic variants in *WT1* are associated with severe nephrotic syndrome that may present as early as the neonatal period. Nephrotic syndrome due to pathogenic variants in *WT1* is almost always resistant to steroids, manifests in childhood, and may coincide with a spectrum of differences of sex development (DSD) (20–22). In particular, XY individuals may demonstrate varying degrees of anomalies of external genitalia (23, 24). This also carries an increased risk of malignancies, including Wilms’ tumor and gonadal tumors that merit close screening (25).

A less common form of *WT1*-related SRNS occurs due to pathogenic variants that disrupt typical splicing of the transcripts of this gene (26, 27). This etiology usually leads to later onset nephrotic syndrome when compared to other forms of *WT1*-related SRNS and may include variable gonadal dysgenesis (28). A hotspot within and near exon 9 of *WT1* has been found to be the most common site of single nucleotide variants that lead to splicing dysregulation underlying this form of podocytopathy (29, 30). Whether splice variants outside of this region could lead to similar disease processes has not been established. In this study, we present the case of an individual with a familial *WT1* variant outside of the hotspot region leading to SRNS and uterovaginal differences. This variant appears to lead to aberrant splicing of *WT1* through a novel mechanism resulting in complete exon 9 skipping. Given that the genetic etiology of individual cases of SRNS and DSDs remains unsolved, this represents an important step in understanding new genetic causes of these clinically challenging disorders.

## Case report

**Figure 1:**
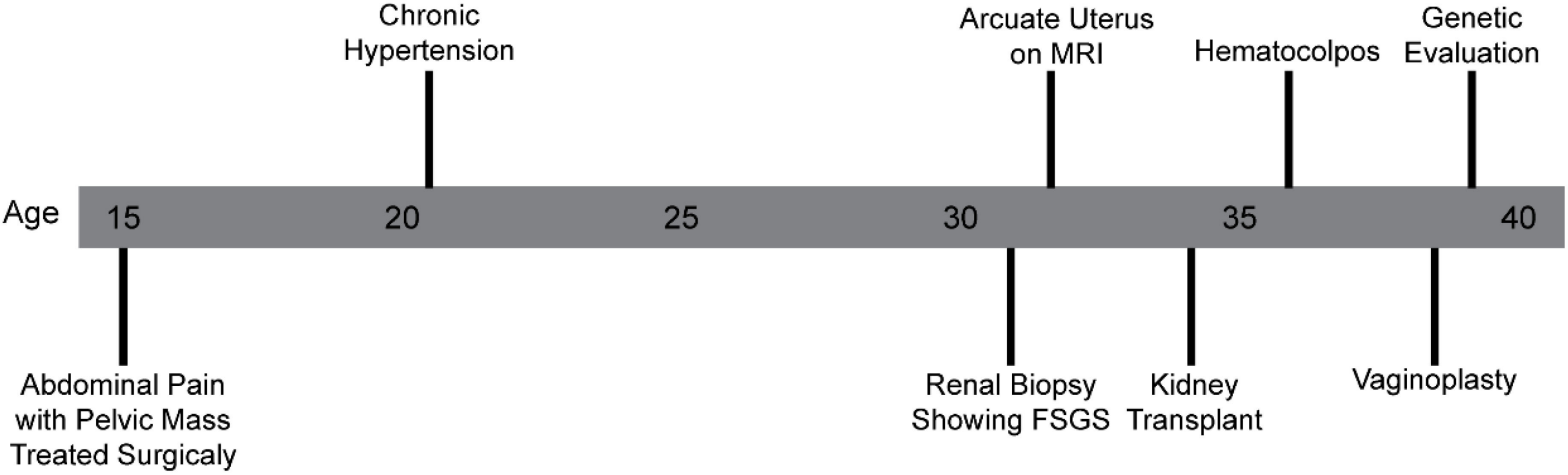
Timeline of the clinical course and evaluation.

A 38-year-old female patient was examined in a subspecialty genetics clinic for differences of sex development (DSD) for the evaluation of the genetic cause of her history of primary ovarian insufficiency and atypical uterovaginal morphology (**Figure 1**).

She was born at term to nonconsanguineous parents who were both of reported Cambodian ancestry. She was in good health through her early adolescence. Around the age of 15, she had increasingly severe abdominal pain and had not yet undergone menarche. This prompted gynecological evaluation that uncovered an unspecified pelvic mass. Her amenorrhea was attributed to an obstructive vaginal anomaly. She underwent a surgical procedure and subsequently experienced regular monthly non-painful menstrual cycles.

She was found to have elevated blood pressure measurements in the hypertensive range in early adulthood. Due to persistence of elevated blood pressure, urinalysis demonstrating nephrotic range proteinuria, and serum creatinine values that were suggestive of a kidney disease, she underwent renal biopsy at age 33 that demonstrated FSGS. Upon progression to end-stage renal disease (ESRD), she underwent a living unrelated renal transplant without excision of her native kidneys. She is maintained on an immunosuppressive regimen of tacrolimus (2 mg in the morning and 1 mg in the evening), mycophenolate mofetil (500 mg twice daily), and prednisone (5 mg daily).

**Figure 2:**
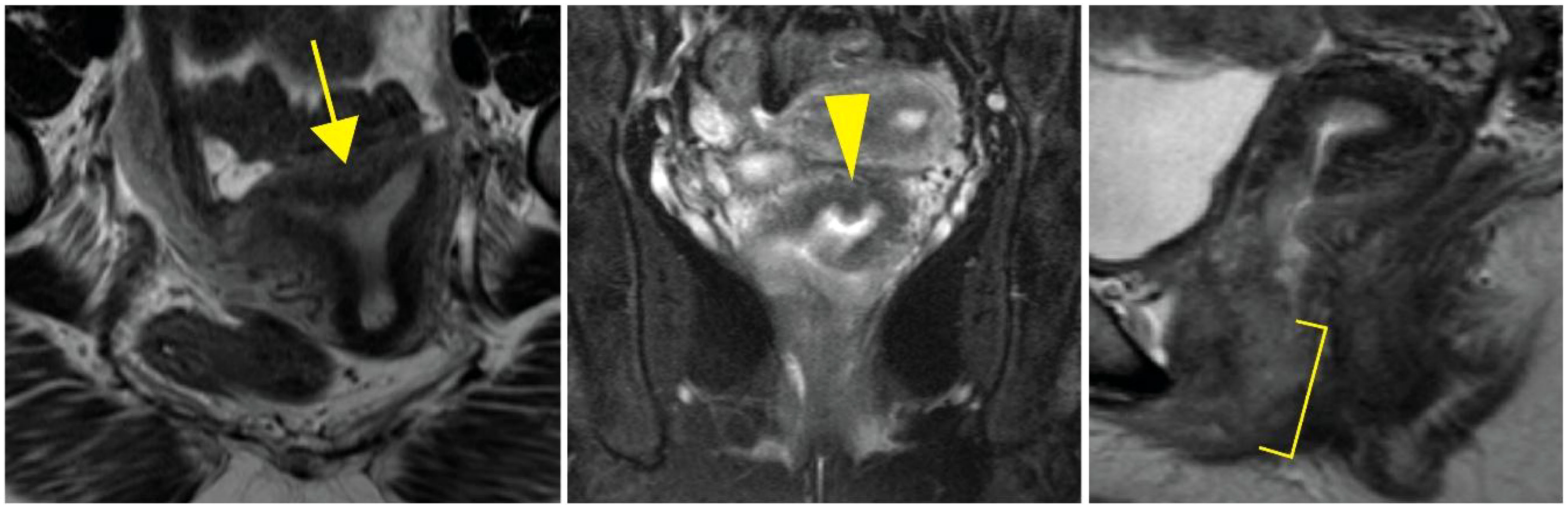
Arcuate uterus in an individual harboring *WT1* c.1338A>C. Axial (left), coronal (middle), and sagittal (right) MRI images demonstrating atypical uterine morphology. The arrow indicates arcuate uterine morphology. The arrowhead indicates a lack of clear uterine septation. The bracket highlights atypical vaginal morphology without a clear vaginal septum.

At age 33, her gynecologic exam was remarkable for atypical morphology of the proximal vaginal canal described as absence of the upper portion of the vagina and an inaccessible cervix. A pelvic MRI demonstrated an arcuate uterus without a definitive vaginal septum (**Figure 2**). Although she was lost to follow-up for approximately 3 years, she represented to care following increasingly frequent hot flashes, irregular menstrual cycles that were then occurring approximately every 2 months, and dysuria. She was diagnosed with distal vaginal atresia with stenosis and underwent vaginoplasty at age 37. Hysteroscopy confirmed arcuate uterine morphology with bilateral tubal ostia visualized. Laboratory values were remarkable at this time for a follicle-stimulating hormone of as high as 46.8 mIU/mL, luteinizing hormone of as high as 389 mIU/mL, and estradiol of as low as 29 pg/mL. She had an ultrasound evaluation with low antral follicle counts of 0 to 1. These findings were consistent with primary ovarian insufficiency; however, her anti-mullerian hormone levels (AMH) were noted to be in the normal range. As part of the evaluation, she underwent further evaluation, including a constitutional karyotype at a 500–650 band resolution that was 46,XX in 20/20 cells.

**Figure 3:**
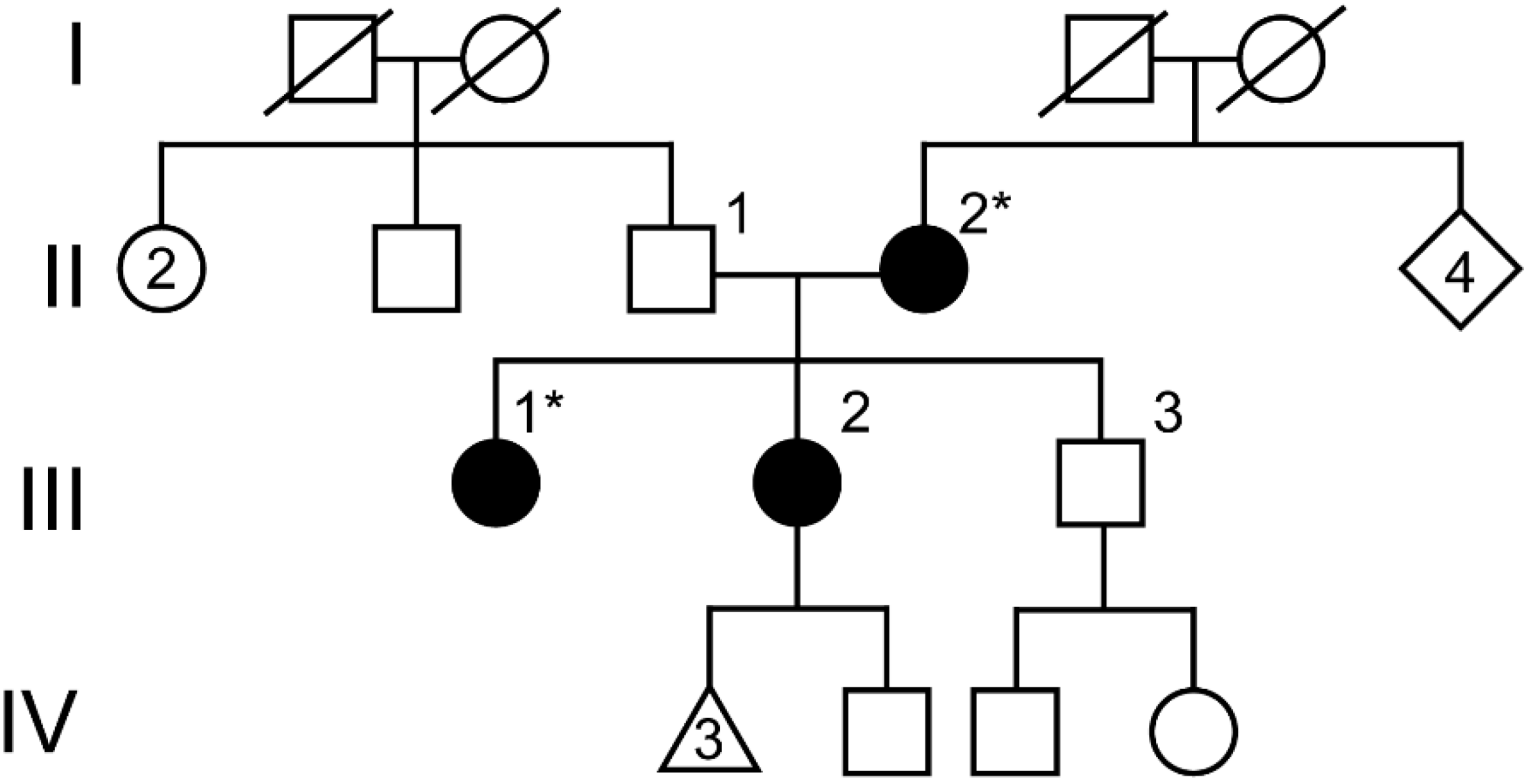
Pedigree of the family with multiple individuals affected with renal disease and uterovaginal differences. The proband (III-1) as well as one full sister (III-2) and their mother (II-2) are affected with renal disease and variable uterine differences (shaded shapes). Asterisks designate individuals that underwent sequencing and were found to harbor the *WT1* c.1338A>C variant (II-2 and III-3). Individuals in generation I are deceased, although their causes of death are not due to renal disease.

Assessment of her family history uncovered a maternal history (II-2) of renal disease in early adulthood requiring renal transplant (**Figure 3**). A full sister also has atypical uterine morphology that was described as bicornate and a history of proteinuria (III-2) (**Figure 3**). Genetic testing was carried out as clinical exome sequencing and copy number variant analysis with the proband and her mother as a comparator. A microarray was not previously completed. This approach allowed for the determination of either copy number or single nucleotide variants that could explain both her and her mother’s history of renal disease. This testing identified the variant: *WT1* g.32413576T>G; NM_024426.4 c.1338A>C; p. = for both the proband and her mother. This variant was classified as a variant of uncertain significance by the reporting reference laboratory. It was not predicted to alter the amino acid sequence of the encoded WT1 protein, although it was suspected possibly to impact splicing.

**Figure 4:**
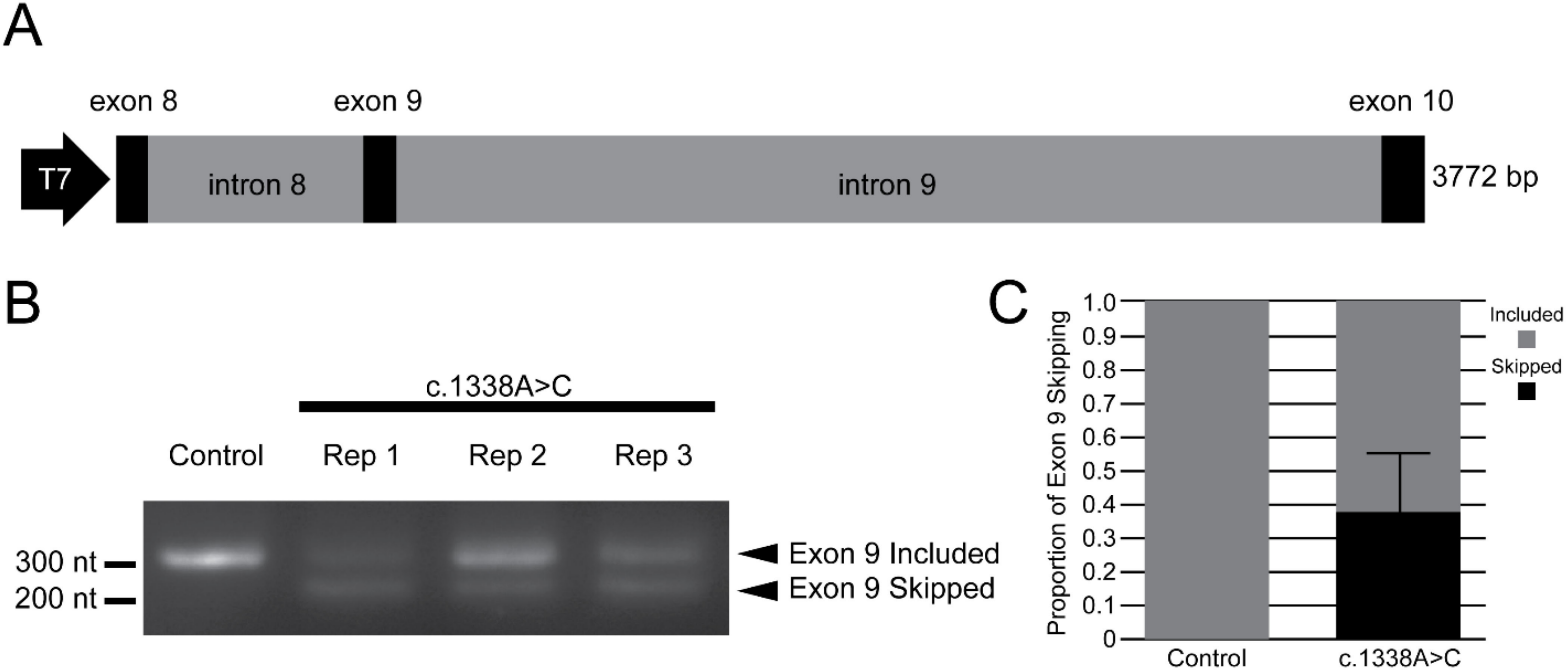
Mini-gene assay demonstrates exon 9 skipping due to *WT1* c.1338A>C. **(A)** Schematic of mini-gene sequence encompassing the 3,772 base pairs that constitute exon 8 through exon 10 of *WT1*. This was present within a pCDNA3.1 vector downstream of a T7 promotor for the expression in HEK293-T cells. Following transfection of either a control pCDNA3.1 vector containing the wild-type mini-gene sequence of exon 8–exon 10 or a site-directed variant with the base change corresponding to c.1338A>C, RNA was collected and reverse transcriptase PCR amplified to detect changes in splicing compared to control. **(B)** Smaller bands present for three biological replicates of the c.1338A>C vector-transfected cells that were not present in controls indicating likely exon 9 skipping due to this variant. **(C)** Quantified relative proportion of exon 9 skipping over the three replicates. Error bar indicates standard deviation.

Based on the absence of this variant from large population databases, including gnomAD and AllofUs, along with the presence of this variant in the two individuals in this family with nephrotic syndrome requiring renal transplant, we considered that this variant may contribute to renal disease and uterovaginal abnormalities seen in the proband. We sought to carry out clinical RNA sequencing that may help clarify the effects of this variant. Yet, due to a lack of readily available tissue that met the requirements of both adequate expression of *WT1* and for which the clinical laboratory had validated protocols for processing, this was not feasible. In lieu of this, we sought to test the impact of the *WT1* variant on splicing *in vitro* through a mini-gene assay that demonstrated *in vitro* evidence of exon 9 skipping (**Figure 4**). This would be predicted to lead to an in-frame transcript lacking a region encoding the third zinc finger domain of WT1 and a disruption of typical WT1 interactions. Importantly, this assay also suggests that a pool of typically spliced *WT1* persists even in the presence of this variant.

## Discussion

The diagnosis of later-onset podocytopathies such as SRNS and/or FSGS is challenging. As many causes such as autoimmune and drug reactions have been implicated in FSGS, adult-onset nephrotic syndrome with histology demonstrating FSGS may not prompt genetic testing as readily as earlier onset disease. Yet, the increased susceptibility of individuals with genetic causes of SRNS to hemodynamic instability, infections, thromboses, renal failure, and in some instances malignancy, merits comprehensive evaluation including genetic testing to identify underlying causes. This is especially true when the family history is suggestive of a familial propensity to syndromic SRNS. This may indeed facilitate addressing the complications of nephrotic syndrome that contribute to significant morbidity and mortality.

In this article, we described an individual with a history of adult-onset SRNS, uterovaginal anomalies, and primary ovarian insufficiency. Based on clinical exome sequencing and research-based assays to evaluate splicing, it seems likely that these differences are due to a variant in *WT1* that affects splicing. This is especially compelling as the variant was also found in her mother, who was a comparator for exome sequencing. This would be a deviation from what appears to be a predominance of *WT1* variants affecting splicing occurring *de novo* (27, 28). Indeed, we hope that our analysis of this variant will aid in the risk stratification of other family members who would be at risk of having inherited this variant. For instance, this may facilitate testing of other family members at risk of having inherited this variant. Family members harboring this variant could thus be more proactively monitored for hypertension and renal disease in a manner that could mitigate the potential systemic effects of these disease processes. Further, the knowledge about the risks posed by inheriting this variant may serve as valuable information for family planning and potentially even various forms of prenatal genetic testing.

Reported pathogenic variants in the *WT1* exon 9 hotspot that have previously been identified to affect splicing in individuals with nephrotic syndrome and DSDs have shown a predominance for 46,XY individuals (21, 22). Yet, *WT1* is understood to play an important role in gonadogenesis in both male and female individuals (31). Variants in *WT1* have been shown to disrupt the interaction of WT1 with β-catenin in a manner that could affect the pro-ovary forming signaling program (32). *WT1* variants may also modify the ability of WT1 to promote the expression of other genes such as *SF1* and *NROB1*, which are important for downstream sex-specific gene expression (31, 33). Thus, aberrant splicing of *WT1* leading to DSD in XX individuals seems plausible. Additionally, variants in *WT1* have been described in individuals with non-syndromic primary ovarian insufficiency (34). Yet, it is unclear why this patient had primary ovarian insufficiency with normal AMH levels. We would expect that as in other instances of *WT1*-related disease, XX findings on karyotype are associated with a low risk of gonadoblastoma and although the risk of Wilms’ tumor remains in XX individuals, this is much more often observed early in life (22).

Although clinical RNA sequencing is becoming an important tool to determine the consequences of variants that may affect splicing or gene expression, this testing modality is limited by tissue-specific gene expression and clinical availability of tissues for testing (35). While clinical RNA sequencing was not feasible in this case, the utility of mini-gene assays to evaluate splicing *in vitro* has been previously demonstrated for similar applications (36, 37). Previous evaluations of clinical RNA sequencing have shown some concordance of clinical testing results with computational tools for predicting the impact of variants on splicing. The splice AI prediction of a delta score of 0.52 for a splice donor site loss was somewhat supportive of a change in typical splicing. This was consistent with our result of exon 9 skipping.

Multiple previously identified variants that affect splicing lead to changes in proportions of *WT1* transcripts containing a region encoding 3 amino acids: lysine–threonine–serine (KTS). This is in contrast to missense, nonsense, and frameshift variants that affect the zinc finger region, as these are predicted to result in an alteration to the specificity of WT1 interacting with the target DNA. The identified variant we reported would be predicted to cause the disease through a mechanism more similar to this latter category of variants. Skipping of *WT1* exon 9 likely results in an in-frame transcript lacking the third zinc finger domain, but the stability and function of the resulting protein are unclear. The third zinc finger domain of WT1 plays a critical role in protein function through the inclusion of a nuclear localization signal that is important for its nuclear import as well as through this domain’s ability to interact with RNA (38, 39). These functions may contribute to the mechanism of the disease, as transgenic mice heterozygous for a variant truncating the third zinc finger recapitulate glomerular mesangial sclerosis, genital defects in males, and an increased incidence in Wilms’ tumor (40). This bolsters our confidence that the mini-gene assay we conducted may be representative of aberrant splicing occurring *in vivo*. Yet, our splicing assay has important limitations. The construct used for expression includes limited sequence context with only three exons and two introns of genetic sequence, and we used a heterologous HEK293-T cell line in which splicing may differ from that of the developing kidney. Tissue-specific splicing differences would similarly not be captured through this assay.

To our knowledge, this report is the first to implicate exon 9 skipping as a likely cause of DSD and SRNS. This adds to the limited number of reports of variants disrupting *WT1* splicing outside of the exon 9 hotspot region.

## Data Availability

The original contributions presented in the study are included in the article/Supplementary Material. Further inquiries can be directed to the corresponding author.
